# Environmental sanitation and the evolution of water, sanitation and hygiene

**DOI:** 10.2471/BLT.21.287137

**Published:** 2022-03-03

**Authors:** Sophie Budge, Argaw Ambelu, Jamie Bartram, Joe Brown, Paul Hutchings

**Affiliations:** a Department of Engineering, School of Civil, Aerospace and Mechanical Engineering, Queen's Building, University Walk, Clifton, University of Bristol, BS8 1QU, England.; b Department of Environmental Health Sciences and Technology, Jimma University, Jimma, Ethiopia.; c Faculty of Engineering and Physical Sciences, University of Leeds, Leeds, England.; d Gillings School of Global Public Health, University of North Carolina, Chapel Hill, United States of America.

The sanitary revolution of the 19th century in much of Europe and North America marked a milestone of public health progress. During this era, transmission dynamics of disease were only crudely appreciated and often specific mechanisms were misunderstood.^1^ Yet expert consensus emerged that infectious disease transmission could be influenced by environmental factors including drainage, proximity to waste, stagnant water, noxious odours, poor air quality, overcrowded housing and other unpleasant conditions.^1^ The practice of engineering and implementation of engineered infrastructure – made possible through industrialization – gave rise to clean water provision, sanitation and changes to the urban landscape that helped transform population health. Together with advances in epidemiology and public health microbiology,^1^ a programme of sanitary reform – encompassing several interventions that came to be known as environmental sanitation – dramatically improved the health and well-being of populations.

The evolving understanding of infectious disease transmission and the interconnected roles of infrastructure and human health have shaped the modern science and practice of water, sanitation and hygiene.^1^ Although the benefits of water, sanitation and hygiene are much broader than its health impacts alone, it is still promoted as a set of health interventions intended to interrupt transmission of infectious diseases originating in excreta. In the past 15 years, scientific advances have refined our approaches to health impact trials and several trials of basic water, sanitation and hygiene interventions have been conducted. These trials have yielded findings of no effect on outcomes such as diarrhoeal disease and child growth.^2^ Commentaries attempting to synthesize the findings from these trials have suggested that many interventions did not reduce pathogen exposure to a threshold that could consistently improve health outcomes.^2^^,^^3^ Authors expressed the need for interventions that radically reduce faecal contamination in the household environment – or an approach described as transformative water, sanitation and hygiene.^2^

Broadly, this approach calls for delivering integrated interventions, including those not previously considered part of water, sanitation and hygiene, of substantial scale and quality to achieve dramatic reductions in enteric pathogen transmission necessary to improve population health.^2^ Emerging definitions of transformative water, sanitation and hygiene are based on outcomes. However, no consensus exists yet on which interventions are needed or at what scale. This ambiguity presents a dilemma for practitioners and policy-makers seeking new strategies for effective environmental health programming. While early notions of transformative water, hygiene and sanitation define the end goal, the sector still needs to resolve the methods for achieving it. A more holistic framing of water, hygiene and sanitation could include improved water quality and quantity, safely managed sanitation, handwashing facilities with soap, the separation of animals and their faeces from living environments, hygiene along the food chain, drainage and solid waste management.^2^ Where these interventions can be delivered effectively at scale, they can become important additions to water, hygiene and sanitation programming to make it more transformative.

The concept of environmental sanitation^4^ captures these elements and others, offering a compelling starting place for operationalizing the transformative approach. The concept draws on the context of historic lessons from the sanitary revolution,^1^ the known range of environmental transmission routes for disease^5^ and emerging lessons from the coronavirus disease 2019 pandemic.^6^ This pandemic demonstrates the importance of multiple controls, from personal responsibilities such as using masks and physical distancing to shared responsibilities such as quarantining and vaccines.^7^ We draw the parallel that effective environmental sanitation interventions are likely to involve both domestic (such as hand hygiene, household water treatment and access to a latrine) and collective (such as community sanitation and waste management, management of animal agriculture, food system safety and regulation, or drainage) elements. In both cases, a layered approach to interventions across many known or suspected transmission pathways is appropriate.

**Fig. 1:**
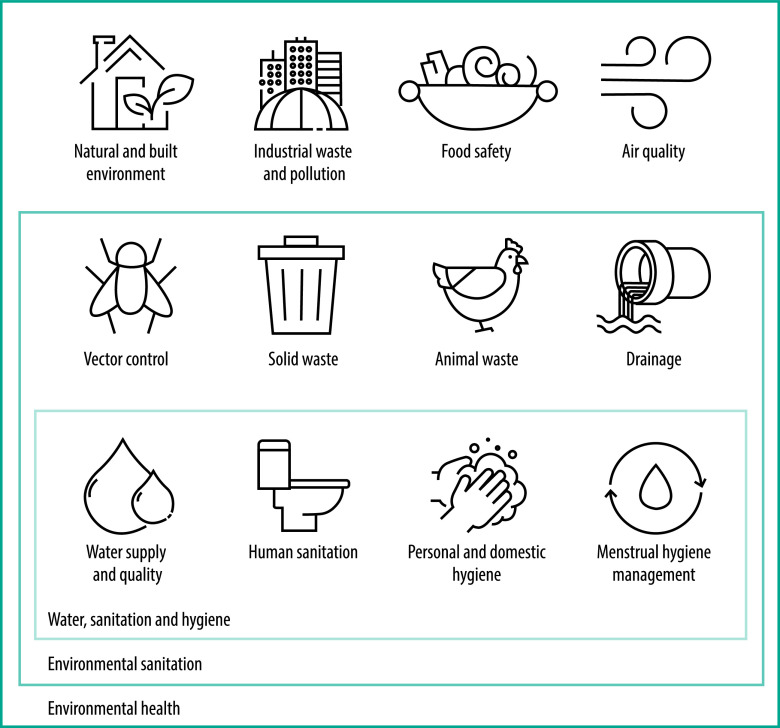
Nested view of environmental interventions for health

Environmental sanitation was an instrumental concept in the founding of the World Health Organization and was defined as the control of all those factors in the physical environment which may exercise a harmful effect on human beings’ physical development, health and survival.^4^ This broad conceptualization of environment and health requires an expansion of the water, sanitation and hygiene space to include vector control, solid waste and animal excreta management and drainage. This definition also involves recognizing related layers of distal environmental health factors (including the natural and built environment, industrial waste and pollution, food safety and air quality) that are increasingly relevant considering population growth, resource use intensification, migration and climate change (Fig. 1). The primary reason for returning to environmental sanitation as a framing concept is that water, sanitation and hygiene has become narrowly focused on – and constrained by – a few pathways of disease transmission that are used to justify a limited set of mostly ineffective interventions at the household level. The global health community needs to consider adjacent factors that determine whether, how and under what circumstances water, sanitation and hygiene can deliver improvements in public health at scale. We need to give greater recognition of the interconnected nature of human health and the natural and built environments and greater attention to community-level exposures. An environmental sanitation perspective would allow interventions to consider the community-level factors that contribute towards infection to sufficiently transform services and improve health outcomes.

Adopting environmental sanitation as a guiding framework will have implications for how to design interventions and how we generate and interpret evidence on effectiveness and impact. First, the scope of trials should include community-level interventions that should take precedence over household or individual-based interventions that are easier to randomize. Recent trials have largely included household-based interventions that place an undue emphasis on individual responsibility for health through behaviour change. For example, the practice of handwashing, although a personal behaviour, is largely determined by social norms that are influenced at the population level and cannot take place without necessary infrastructure. In settings without such infrastructure but with high levels of faecal contamination, the practice of handwashing may not be able to influence overall exposures, no matter how diligent households might be^8^ – placing an unfair burden on individuals. The lack of effect from household-level interventions has sometimes been interpreted as a criticism of personal behaviours rather than supporting infrastructure, which must change. We should focus on a more nuanced spatial and temporal understanding of disease control; transmission pathways do not act in isolation.

Second, the application of sophisticated tools in environmental health microbiology increases the ability to track the movement of pathogens in the environment, bringing with them the potential to revolutionize understanding in this area. Using these tools to advance the evidence base will require overcoming the reductionism in measurement approaches that has characterized trials assessing single transmission pathways and moving to more complex and multilayered assessments of transmission. Exposure measurement at scale may shift the locus of environmental sampling and analysis from households to communities.^9^^–^^11^ Such a shift aligns with longer-term evidence indicating that a multifaceted and sustained change in infrastructure and service provision,^3^ and emphasis on changes to population-level exposures, are required to track changes at this scale and over appropriate time frames.

Third, we should lower expectations of distal outcome measures such as child growth within water, sanitation and hygiene impact trials. Such outcomes involve highly complex phenomena and require working across sectors in both intervention delivery and the design of impact trials. Only when progress is made across multiple fronts – for instance nutrition, infrastructure pertaining to housing, water, faecal waste, solid waste, air and more – might we achieve the environmental hygiene threshold necessary to support such outcomes.^3^ Further research into such a saturation point at which inventions reach necessary levels^12^ for such improvements is important. Where appropriate, trials should focus on proximal outcome measures that are balanced between domestic and public spheres, reflecting the reality of where people spend time and where exposures may be relevant.

Water, sanitation and hygiene as practised since the middle of the 20th century has become an imperfect fit with what we now know about enteric disease transmission and how to control it. Pathogens are transmitted along interconnected pathways that are far more nuanced than the traditional F-diagram (describing the faecal-oral route of disease transmission) and, in endemic settings, interventions addressing single pathways are unlikely to deliver health benefits. Considering how the water, sanitation and hygiene concept and its operational framework must evolve to bring about improvements to global public health has become necessary. Water, sanitation and hygiene is primarily useful within the long-established framing of environmental sanitation, and not as a rigid, sometimes insular sector that fails to adapt to a more sophisticated understanding of the problem space. As illustrated in Fig. 1, we must recognize that most environmental health interventions involve complex relationships between different layers of influence, with systemic interactions between them, and that all these layers need to be considered.^11^ We fully support the pursuit of more effective water, sanitation and hygiene as captured in the transformative approach, and we believe that environmental sanitation provides a compelling operational framing to achieve the promised goal of improved population health and well-being. 
